# Proactive versus Rank-Down Topical Corticosteroid Therapy for Maintenance of Remission in Pediatric Atopic Dermatitis: A Randomized, Open-Label, Active-Controlled, Parallel-Group Study (Anticipate Study)

**DOI:** 10.3390/jcm11216477

**Published:** 2022-10-31

**Authors:** Koji Kamiya, Hidehisa Saeki, Yoshiki Tokura, Shigemi Yoshihara, Junichi Sugai, Mamitaro Ohtsuki

**Affiliations:** 1 Department of Dermatology, Jichi Medical University, 3311-1 Yakushiji, Shimotsuke 329-0498, Tochigi, Japan; 2 Department of Dermatology, Nippon Medical School, 1-1-5 Sendagi, Bunkyo-ku 113-8602, Tokyo, Japan; 3 Allergic Disease Research Center and Department of Dermatology & Skin Oncology, Chutoen General Medical Center, 1-1 Shobugaike, Kakegawa 436-8555, Shizuoka, Japan; 4 Department of Pediatrics, Dokkyo Medical University, 880 Kitakobayashi, Mibu 321-0293, Shimotsuga, Tochigi, Japan; 5 Sugai Dermatology Parkside Clinic, 6-7-7 Motoimaizumi, Utsunomiya 321-0954, Tochigi, Japan

**Keywords:** atopic dermatitis, pediatric, proactive therapy, rank-down therapy, topical corticosteroids

## Abstract

Topical corticosteroids are used as first-line treatment for atopic dermatitis (AD). Regarding the maintenance of remission achieved by topical corticosteroids, no previous studies have compared proactive therapy with rank-down therapy. We compared their efficacy and safety in Japanese children with moderate to severe AD. Patients who had achieved remission with a very strong topical corticosteroid were randomized to 4-week maintenance treatment with either intermittent use of the same drug (proactive therapy) or daily use of a strong topical corticosteroid for 1 week followed by daily use of a medium-potency topical corticosteroid for 3 weeks (rank-down therapy); 49 patients were randomized (proactive therapy, *n* = 24; rank-down therapy, *n* = 25). During maintenance treatment, the relapse rate was 8.33% in the proactive therapy group and 20.0% in the rank-down therapy group (*p* = 0.0859). The mean (±standard deviation) itching score on a numerical rating scale in the rank-down therapy group increased significantly from 2.5 ± 1.9 to 3.6 ± 2.6 (*p* = 0.0438). Adverse events occurred in 2 patients receiving proactive therapy and 3 patients receiving rank-down therapy. Proactive therapy appears to be as safe as rank-down therapy and may be more effective for itch in pediatric AD in remission.

## 1. Introduction

Atopic dermatitis (AD) is a relapsing and remitting disease characterized by itchy eczema. Most patients with AD have a previous and/or family history of allergic disorders and atopic predispositions, such as an increased liability to produce IgE antibodies [1]. In the US, the prevalence of AD in people aged 17 years or younger has been reported to be 12.5%, with the disease affecting 14.2%, 13.1%, and 10.9% of those aged 0 to 4 years, 5 to 9 years, and 10 to 17 years, respectively [2]. A survey in Japanese elementary schoolchildren revealed a similar prevalence of pediatric AD to those in the US, with a rate of 11.2% overall, 11.8% in first-grade students, and 10.5% in sixth-grade students [3].

Japanese, US, and European guidelines recommend topical corticosteroids (TCS) as a first-line treatment for AD [1,4,5]. In Japan, TCS are ranked by their potencies into Group 1 (strongest class), 2 (very strong class), 3 (strong class), 4 (medium class), and 5 (weak class); according to the guideline, a TCS should be chosen from the most appropriate group according to the disease severity and affected site [1].

TCS can have both systemic and local adverse effects [1]. Systemic adverse effects, such as suppression of adrenal function, are usually associated with the use of more potent TCS, and optimization of the potency and dosage of these drugs could minimize the risk of systemic adverse effects [6,7]. Local adverse effects of TCS include vasodilation and skin atrophy; such effects can be prevented by reducing the dosing frequency and can resolve with appropriate treatment [6,8,9].

Proactive (PA) therapy was devised for the management of AD with frequent relapses. In this approach, a high-potency TCS of the same rank as the initial treatment is used intermittently as maintenance treatment after remission is achieved. PA therapy was reported to reduce the risk of both AD relapse and adverse effects of TCS treatment [10,11,12,13,14,15,16]. PA therapy also provided significantly better rash control and quality of life compared with reactive therapy (on-demand TCS use upon relapse) in pediatric patients with AD aged 3 months to 7 years [2]. Japanese guidelines also recommend considering the use of PA therapy for management of AD with frequent relapses [1]. Nonetheless, the efficacy of PA therapy with intermittent TCS use has been established only in comparison with placebo or moisturizer treatment alone [17,18,19,20,21,22,23] or with reactive therapy upon relapse [24], and no previous studies have compared PA with rank-down (RD) therapy, in which a less potent TCS is used after remission induction. Furthermore, although TCS of all potencies can be used in patients of all ages, physicians usually consider modifying the duration of TCS treatment in infants and children because they are more likely to respond to short-term treatment [1]. Therefore, in the present study, we evaluated the efficacy and safety of PA therapy compared with RD therapy in Japanese children with moderate to severe AD, who have a higher risk of exacerbations than patients with mild disease.

## 2. Materials and Methods

### 2.1. Study Design

This was a multicenter, randomized, open-label, active-controlled, parallel-group study to investigate the efficacy and safety of PA therapy versus RD therapy in male and female pediatric patients with moderate to severe AD. The study was conducted from June 2019 to December 2021 at 21 institutions in Japan.

**Figure 1:**
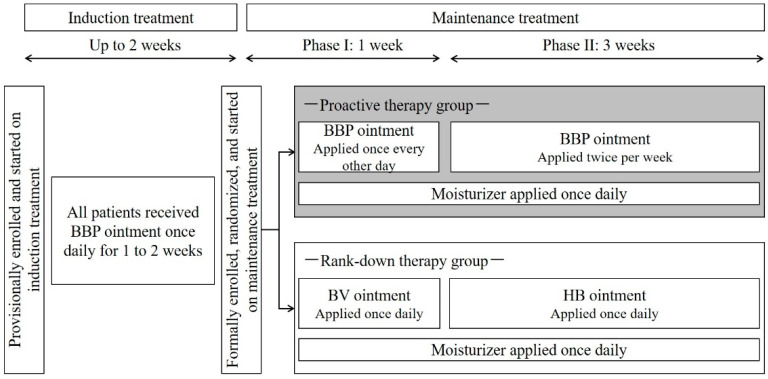
Study design. Abbreviations: BBP, betamethasone butyrate propionate; BV, betamethasone valerate; HB, hydrocortisone butyrate.

The study consisted of a 2-week induction period followed by a 4-week maintenance period (Figure 1). During the induction period, provisionally enrolled patients were treated with betamethasone butyrate propionate (BBP ointment), a very strong TCS, once daily for at least 1 week and up to 2 weeks. Patients who achieved remission during the induction period (defined as an Investigator Global Assessment (IGA) score ≤ 2) were eligible for maintenance treatment and were randomized with an electronic data capture system (stratified for baseline IGA score and age) to receive either PA therapy (PA group) or RD therapy (RD group). PA therapy comprised intermittent treatment with BBP ointment, and RD therapy comprised daily treatment with betamethasone valerate (BV) ointment, a strong TCS, followed by hydrocortisone butyrate (HB) ointment, a medium-class TCS.

Maintenance therapy was administered in 2 phases, phase I (1 week) and phase II (3 weeks; Figure 1). In the PA group, BBP ointment was applied to the area where a rash had been present at the start of induction treatment; the area was treated once a day every other day during phase I and twice per week during phase II. In the RD group, ointment was applied once daily to the area where a rash was currently present; BV ointment was applied during phase I, and HB ointment was applied during phase II. In both groups, patients also received daily treatment with a moisturizer or petrolatum for skin care throughout the maintenance period.

While participating in the study, patients were prohibited from using biologics, systemic corticosteroids and immunosuppressants, live vaccines, and non-study topical anti-inflammatory agents. Concomitant use of antihistamines, antiallergic agents, and tacrolimus ointment or TCS for head/neck lesions was permitted only if their use had been started before enrollment and was to be continued at the same dose throughout the study.

The study was conducted with due consideration to the rights of individual study participants, in accordance with the ethical principles that have their origin in the Declaration of Helsinki (latest revision in October 2013) and in compliance with the Clinical Trials Act and the Enforcement Regulations of the Act. All participants and their legal representatives received sufficient explanations about the study and gave written informed assent and consent to the study prior to enrollment. The study protocol was approved by the Jichi Medical University Central Clinical Research Ethics Committee. The study was registered at the Japan Registry of Clinical Trials (jRCTs031190047).

### 2.2. Patients

To be eligible for the study, patients had to meet the following inclusion criteria: (1) Japanese ethnicity and a diagnosis of AD according to the Japanese Dermatological Association (JDA) Definition and Diagnostic Criteria for AD [1]; (2) aged 6 to 15 years; (3) IGA score of 3 or more; (4) previous use of a strong or very strong TCS; and (5) provision of written informed assent or consent.

The main exclusion criteria included the following: (1) presence of any bacterial, fungal, spirochetal, or viral skin infection or ectoparasitic skin disease; (2) presence of any ulcer (except for Bechet’s disease), deep burn, or frostbite of the second degree or higher; (3) co-existence of any active infection in the area where the study drug was to be applied; (4) concomitant Kaposi’s varicelliform eruption, scabies, molluscum contagiosum, impetigo contagiosa, psoriasis, disorders presenting with ichthyosiform erythroderma, collagen disease, or any skin disorder on the area where the study drug was to be applied that could affect evaluation; (5) use of systemic adrenocortical steroids, strongest-class TCS, systemic immunosuppressants, or live vaccines within 28 days before the time of informed assent/consent; and (6) phototherapy performed within 28 days before the time of informed assent/consent.

### 2.3. Study Endpoints

The primary efficacy endpoint was the relapse rate during the maintenance period, whereby relapse was defined as an IGA score of 3 or more.

Secondary outcome measures were as follows: (1) duration of remission (time to relapse); (2) IGA score; (3) modified Severity Scoring of Atopic Dermatitis (SCORAD) severity and disease extent scores; and (4) symptom scores assessed on a numerical rating scale (NRS). The SCORAD disease extent score was calculated as the sum of the percentages of the body surface areas affected (assessed on a 4-level scale of 0, 1/3, 2/3, and 3/3) in the following 8 body segments: head/neck (9%), anterior trunk (18%), groin (1%), posterior trunk (18%), left arm (9%), right arm (9%), left leg (18%), and right leg (18%). An 11-point NRS ranging from 0 (asymptomatic) to 10 (greatest intensity) was used to rate the severity of itching and sleep disturbance; for each symptom, the mean of the scores on the 3 days prior to the visit was calculated. In patients who wished to undergo measurement of AD-related biomarkers, total serum IgE, peripheral blood eosinophil count, serum lactate dehydrogenase (LDH), and serum thymus and activation-regulated chemokine (TARC) were also measured.

For the safety evaluation, adverse events (AEs) during the study period were recorded. An AE was defined as any untoward medical event that occurred during the period from the start of induction treatment until 4 weeks after the start of maintenance treatment (or the time of study termination). In patients who wished to undergo monitoring of the effects of TCS on the hypothalamus–pituitary–adrenocortical axis, serum levels of cortisol and adrenocorticotropic hormone (ACTH) were measured.

### 2.4. Statistical Analyses

Based on the assumption that the relapse rate during PA therapy was 27%, a sample size of 35 participants per group was calculated as the least number of participants needed to show a relapse rate of less than 50% in the PA group with a statistical power of 80% at an alpha error rate of 5%.

The efficacy and safety of maintenance treatment were compared between the two groups. Efficacy was evaluated in the full analysis set (FAS), which included all patients except those with a significant protocol violation and those who did not receive the study treatment. The safety analysis set included patients enrolled in the study who started treatment and received all or part of the study treatment.

Descriptive data are expressed as *n* (%), mean (±standard deviation [SD]), and/or median (range). Categorical variables were compared with Fisher’s direct probability test, and continuous variables were compared with the paired *t* test or Wilcoxon rank-sum test. The relapse rate was estimated by the Kaplan–Meier method and compared between the two groups with the log-rank test. A two-sided *p* value of less than 0.05 was considered statistically significant.

## 3. Results

### 3.1. Patient Characteristics

**Figure 2:**
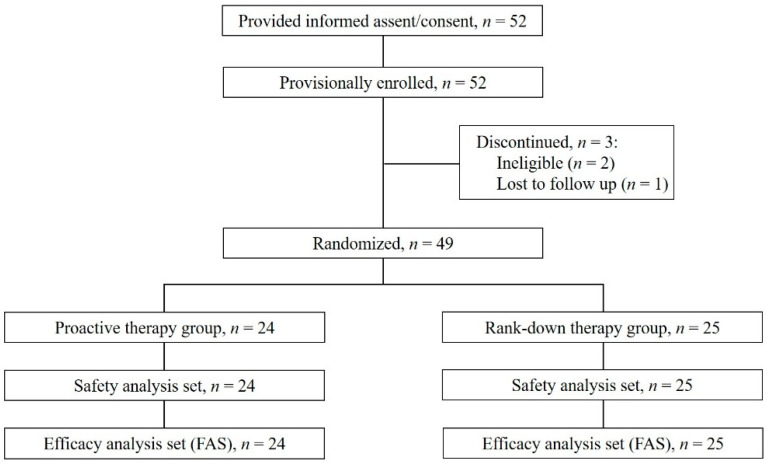
Flowchart of study participants. Abbreviation: FAS, full analysis set.

Of the 52 patients who provided informed assent/consent, 3 were not eligible for the study. The remaining 49 patients were enrolled and randomized to the PA group (*n* = 24) or RD group (*n* = 25; Figure 2). Patient characteristics are shown in Table 1. Just over half of the whole group were boys (51.0%); the mean age was 9.3 ± 2.2 years, and the mean weight was 32.4 ± 12.5 kg. The duration of AD was less than 5 years in at least half the patients (54.2%), including 12 (50.0%) in the PA group and 14 (58.3%) in the RD group (*n* = 24, excluding one patient with an unknown duration). All patients had been previously treated for AD, and in both groups, the majority of participants had a family history of AD (*n* = 44, 89.8%). Itching was reported by 44 patients (89.8%). Erythema was the most common acute manifestation of AD (*n* = 35, 71.4%), and weeping erythema/lichenification was the most common chronic manifestation (*n* = 35, 71.4%). The two groups were well balanced with regard to baseline characteristics and disease status.

### 3.2. Relapse Rate

**Figure 3:**
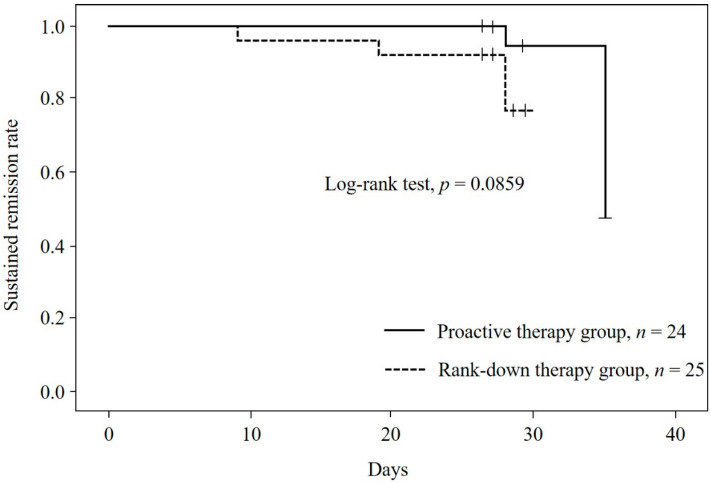
Kaplan–Meier estimate of sustained remission rate with proactive therapy versus rank-down therapy.

The relapse rate was numerically lower in the PA group than in the RD group, although the difference was not statistically significant (8.33% (95% CI, 1.03–27.00%) versus 20.0% (95% CI, 6.83–40.70%); *p* = 0.0859; Figure 3).

### 3.3. Secondary Outcome Measures

The secondary outcome measures are shown in Table 2. The mean duration of remission was similar in both groups (28.3 ± 2.3 days in the PA group and 26.9 ± 4.3 days in the RD group).

In both groups, the IGA score, modified SCORAD severity and disease extent scores, and NRS symptom scores were lower at the start of maintenance treatment (baseline) than at the start of induction treatment. At 4 weeks after the start of maintenance treatment, both NRS symptom scores were unchanged from baseline in the PA group, but the NRS itching score had increased significantly in the RD group, from 2.5 ± 1.9 at baseline to 3.6 ± 2.6 (*p* = 0.0438). No significant change from baseline was found in any other score, and no significant differences were found between the two groups at baseline and 4 weeks.

Table S1 shows the mean blood test results from a subset of patients in the two groups (*n* = 1 to 4 per parameter). At baseline, the mean serum IgE titer was above the upper limit of normal in both groups (1598 ± 1529 IU/mL (*n* = 4) in the PA group; 1590 ± 2359 IU/mL (*n* = 3) in the RD group). At 4 weeks, no significant change from baseline was observed in the peripheral blood eosinophil count, serum LDH, or serum TARC in either group. For all AD-related biomarkers, no significant differences were found between the two groups in the mean values at baseline and 4 weeks. Although the peripheral blood eosinophil count remained relatively unchanged in the PA group, it increased in the RD group, from 313.4 ± 417.5/μL (*n* = 3) to 662.0 ± 619.4/μL (*n* = 2). Serum TARC showed a greater increase in the RD group than in the PA group, but none of the differences were significant (918.0 ± 664.2 pg/mL (*n* = 3) to 1689.0 ± 1156.8 pg/mL (*n* = 2)).

Individual blood test data (Table S2) indicated that the increases in mean peripheral blood eosinophil count and serum TARC during RD therapy were primarily attributable to the increases observed in Patient 5, who showed the greatest increases in the respective parameters, from 790/μL to 1100/μL and from 1646 pg/mL to 2507 pg/mL.

### 3.4. Safety

AEs occurred in 2 patients in the PA group (8.3%) and 3 patients in the RD group (12.0%) (Table 3). Skin papilloma was reported in both patients in the PA group (8.3%); in the RD group, the reported AEs were upper respiratory tract infection in 1 patient (4.0%), Kaposi’s varicelliform eruption in 1 patient (4.0%), and cutaneous ulceration in 1 patient (4.0%). None of the AEs were serious. In both groups, the serum cortisol and ACTH levels showed no significant change from baseline to 4 weeks and did not differ significantly between the two groups at either time point (Table S1). Individual data on serum cortisol and ACTH levels also showed no notable changes from baseline at 4 weeks (Table S2).

## 4. Discussion

We evaluated the efficacy and safety of PA versus RD maintenance TCS therapy in Japanese children with moderate to severe AD and found a numerically lower relapse rate with PA therapy than with RD therapy (8.33% (95% CI, 1.03–27.00%) versus 20.0% (95% CI, 6.83–40.70%); *p* = 0.0859; Figure 3). Previous parallel-group controlled studies of 16- to 20-week maintenance treatment reported relapse rates of 5.6% to 19.0% during PA therapy with topical methylprednisolone or fluticasone compared with 32.2% to 64.0% with placebo or moisturizer treatment alone [17,18,19]. Although the present study used a different protocol from the previous studies with regard to the TCS used and duration of observation, the relapse rate observed during PA therapy in the present study was within the range of values reported in the previous studies. The relapse rate during RD therapy was reasonably expected to be lower in the present study than in the control arms of the previous studies, and our findings were consistent with this expectation.

In the present study, the NRS itching score at 4 weeks showed no significant change from baseline in the PA group (2.2 ± 2.0 to 2.8 ± 2.2, *p* = 0.1877) but increased significantly in the RD group (2.5 ± 1.9 to 3.6 ± 2.6, *p* = 0.0438) (Table 2). This difference suggests that PA therapy may be more effective than RD therapy in controlling itching associated with AD due to applying method and corticosteroids potency. Because relapse of AD was defined as a score of ≥3 on the IGA scale (a measure of skin lesions), the increase in the NRS itching score observed in the RD group does not indicate AD relapse. However, because itching due to AD makes patients want to scratch, which can trigger exacerbations of skin symptoms [25,26,27,28], the increased intensity of itching may have contributed to the numerically higher relapse rate in the RD group.

To the best of our knowledge, no previous controlled studies have compared PA and RD therapy. Therefore, the present study provided clinically meaningful results in that it showed a tendency towards a higher likelihood of maintaining remission with PA therapy than with RD therapy and an increased intensity of itching only with RD therapy. Nonetheless, these results were obtained under the specific experimental conditions used in the present study, in which patients randomized to the PA group used a very strong TCS (BBP ointment) intermittently, whereas those randomized to the RD group used a strong TCS (BV ointment) daily for 1 week and then a medium-class TCS (HB ointment) daily for 3 weeks. Further randomized controlled studies should be conducted to determine whether other PA and RD therapies show greater differences.

The biomarkers of peripheral blood eosinophil count, serum LDH, and serum TARC reflect the severity of AD [1,29,30]. In the present study, the levels of these markers were measured only in a small number of patients who wished to undergo the tests. One patient in the RD group had a high peripheral blood eosinophil count (1100/μL) and serum TARC level (2507 pg/mL) at 4 weeks, although the values of both parameters were high also at baseline (790/μL and 1646 pg/mL, respectively) (Table S2). In some patients with AD who appear to be in remission and have normal skin findings, inflammatory cells may remain in the skin tissue and can contribute to a relapse of skin inflammation [31,32]. In the above-mentioned patient, the high baseline levels of these biomarkers indicate that the patient might have been at high risk of developing a relapse of skin inflammation; the increases at 4 weeks may have occurred even if the patient had been maintained on PA therapy, and it is difficult to determine whether the results are definitely related to differential effects of the maintenance strategies. In clinical practice, it may be advisable for physicians to consider switching to maintenance TCS therapy (either PA or RD therapy) only in patients with sufficiently low levels of biomarkers (e.g., TARC).

The incidence of AEs was similar between the two treatment groups (PA group, 8.3% (*n* = 2/24); RD group, 12.0% (*n* = 3/25); Table 3). The serum levels of cortisol and ACTH were measured only in a small number of patients, but the results showed no notable changes, indicating that neither of the maintenance TCS therapies altered hypothalamus–pituitary–adrenocortical axis activity. Thus, the present study did not reveal any apparent differences in the safety of PA and RD therapies.

The study has three limitations. First, the study findings were obtained under strict experimental conditions (e.g., the TCS used, their dosage, and duration of observation) and are not generalizable to other comparisons of PA and RD therapies. Second, the Infants and Toddlers Dermatology Quality of Life (InToDermQoL) was recently developed [33] and QoL assessment is also required in clinical practice. Third, the sample size was small, and the study was not sufficiently powered to show superiority of one therapy over the other. These limitations should be taken into account when interpreting the results of this study.

## 5. Conclusions

In conclusion, in children with moderate to severe AD in remission, PA therapy with a very strong TCS may prevent the worsening of itching (as measured by the NRS itching score) observed with RD therapy. The two maintenance strategies appear to be equally safe. These findings suggest that PA therapy may be preferable to RD therapy for itch reduction in pediatric patients with moderate to severe AD in remission.

## Figures and Tables

**Table 1:** Patient characteristics.

	Total (*n* = 49)	Proactive Therapy Group (*n* = 24)	Rank-Down Therapy Group (*n* = 25)
Sex, *n* (%), male	25 (51.0%)	11 (45.8%)	14 (56.0%)
Age, mean ± SD, y	9.3 ± 2.2	9.1± 2.2	9.5 ± 2.2
Height, mean ± SD, cm	134.0 ± 13.4	133.4 ± 14.6	134.5 ± 12.5
Weight, mean ± SD, kg	32.4 ± 12.5	31.0 ± 12.4	33.7 ± 12.8
Duration of AD, *n* (%) ^†^			
<5 years	26 (54.2%)	12 (50.0%)	14 (58.3%)
≥5 years, <10 years	13 (27.1%)	7 (29.2%)	6 (25.0%)
≥10 years	9 (18.8%)	5 (20.8%)	4 (16.7%)
Family history, *n* (%)			
No	5 (10.2%)	2 (8.3%)	3 (12.0%)
Yes	44 (89.8%)	22 (91.7%)	22 (88.0%)
Previous medical history, *n* (%)			
No	36 (73.5%)	18 (75.0%)	18 (72.0%)
Yes	13 (26.5%)	6 (25.0%)	7 (28.0%)
Concomitant illnesses, *n* (%)			
No	16 (32.7%)	8 (33.3%)	8 (32.0%)
Yes	33 (67.3%)	16 (66.7%)	17 (68.0%)
Prior anti-AD therapy, *n* (%)			
No	0	0	0
Yes	49 (100.0%)	24 (100.0%)	25 (100.0%)
Itching, *n* (%)			
No	5 (10.2%)	2 (8.3%)	3 (12.0%)
Yes	44 (89.8%)	22 (91.7%)	22 (88.0%)
Acute skin lesions, *n* (%)			
None	12 (24.5%)	6 (25.0%)	6 (24.0%)
Erythema	35 (71.4%)	17 (70.8%)	18 (72.0%)
Weeping erythema	8 (16.3%)	2 (8.3%)	6 (24.0%)
Papule	19 (38.8%)	8 (33.3%)	11 (44.0%)
Serous papule	5 (10.2%)	1 (4.2%)	4 (16.0%)
Scale	14 (28.6%)	5 (20.8%)	9 (36.0%)
Scab	9 (18.4%)	4 (16.7%)	5 (20.0%)
Chronic skin lesions, *n* (%)			
None	7 (14.3%)	3 (12.5%)	4 (16.0%)
Weeping Erythema/lichenification	35 (71.4%)	17 (70.8%)	18 (72.0%)
Prurigo	7 (14.3%)	2 (8.3%)	5 (20.0%)
Scale	20 (40.8%)	7 (29.2%)	13 (52.0%)
Scab	13 (26.5%)	5 (20.8%)	8 (32.0%)

† One patient with an unknown duration of disease in the rank-down therapy group was excluded from the calculation (*n* = 24 for the group). Abbreviations: AD, atopic dermatitis; SD, standard deviation.

**Table 2:** Atopic dermatitis-related clinical outcome measures.

	Proactive Therapy Group (*n* = 24)	*p* ^†^: vs. Start of Maintenance Treatment	Rank-Down Therapy Group (*n* = 25)	*p* ^†^: vs. Start of Maintenance Treatment	*p* ^‡^: Between-Group Comparison
Duration of remission, mean ± SD, Days	28.3 ± 2.3		26.9 ± 4.3		-
IGA score, mean ± SD					
At start of induction treatment	3.0 ± 0.2		3.1 ± 0.3		-
At start of maintenance treatment	1.5 ± 0.5	-	1.8 ± 0.4	-	*p* = 0.0618
At 4 weeks	1.4 ± 0.8	*p* = 0.5637	1.6 ± 0.9	*p* = 0.3711	*p* = 0.5441
Modified SCORAD score, mean ± SD					
Severity score					
At start of induction treatment	48.7 ± 19.6		46.6 ± 18.3		-
At start of maintenance treatment	16.2 ± 6.2	-	21.5 ± 10.4	-	*p* = 0.1025
At 4 weeks	17.3 ± 16.0	*p* = 0.4579	25.4 ± 18.2	*p* = 0.3100	*p* = 0.1136
Disease extent score					
At start of induction treatment	43.2 ± 13.7		37.2 ± 12.4		-
At start of maintenance treatment	25.5 ± 10.2	-	26.0 ± 11.2	-	*p* = 0.5174
At 4 weeks	26.3 ± 11.6	*p* = 0.7514	25.9 ± 14.2	*p* = 0.8079	*p* = 0.9275
NRS symptom score, mean ± SD					
Itching score					
At start of induction treatment	5.4 ± 2.4		5.1 ± 2.1		-
At start of maintenance treatment	2.2 ± 2.0	-	2.5 ± 1.9	-	*p* = 0.5491
At 4 weeks	2.8 ± 2.2	*p* = 0.1877	3.6 ± 2.6	*p* = 0.0438	*p* = 0.2706
Sleep disturbance score					
At start of induction treatment	1.7 ± 2.4		2.6 ± 3.1		-
At start of maintenance treatment	1.2 ± 1.8	-	1.1 ± 1.6	-	*p* = 0.9299
At 4 weeks	1.1 ± 2.1	*p* = 0.8571	1.5 ± 1.9	*p* = 0.3101	*p* = 0.2619

*p* †: Wilcoxon signed rank-sum test, *p* ‡: Wilcoxon rank-sum test. Abbreviations: IGA, Investigator Global Assessment; NRS, numerical rating scale; SCORAD, Severity Scoring of Atopic Dermatitis; SD, standard deviation.

**Table 3:** Adverse events observed during the study period.

	Total	Proactive Therapy Group	Rank-Down Therapy Group
Safety analysis set, *n*	49	24	25
Patients with any adverse event, *n* (%)	5 (10.2%)	2 (8.3%)	3 (12.0%)
Adverse events, *n* (%)			
Infections and infestations	2 (4.1%)	0	2 (8.0%)
Upper respiratory tract infection	1 (2.0%)	0	1 (4.0%)
Infections and infestations, other (Kaposi’s varicelliform eruption)	1 (2.0%)	0	1 (4.0%)
Neoplasms benign, malignant and unspecified (incl cysts and polyps)	2 (4.1%)	2 (8.3%)	0
Skin papilloma	2 (4.1%)	2 (8.3%)	0
Skin and subcutaneous tissue disorders	1 (2.0%)	0	1 (4.0%)
Cutaneous ulceration	1 (2.0%)	0	1 (4.0%)

## Data Availability

Not applicable.

## Supplementary Materials

The following supporting information can be downloaded at: https://www.mdpi.com/article/10.3390/jcm11216477/s1, Table S1: Blood test results: Group mean data; Table S2: Blood test results: Individual data.
