# Supplementary material for: Proactive versus Rank-Down Topical Corticosteroid Therapy for Maintenance of Remission in Pediatric Atopic Dermatitis: A Randomized, Open-Label, Active-Controlled, Parallel-Group Study (Anticipate Study)
Source: J Clin Med. 2022 Oct 31;11(21):6477. doi: 10.3390/jcm11216477 (PMC9658234; doi:10.3390/jcm11216477)
Supplement: Supplementary file 1 [file jcm-11-06477-s001.zip › SuppInfo_Supplemental Table S1.pdf]

Supplemental Table S1. Blood test results: Group mean data.

	Proactive therapy group (n = 24)	p†: vs. start of maintenance treatment	Rank-down therapy group (n = 25)	p†: vs. start of maintenance treatment	p‡: Between-group comparison
Atopic dermatitis-related biomarkers					
Serum total IgE, mean ± SD, IU/mL					
At start of maintenance treatment	1598 ± 1529 (n = 4)	-	1590 ± 2359 (n = 3)	-	-
Peripheral blood eosinophil count, mean ± SD, /µL					
At start of maintenance treatment	512.0 ± 384.6 (n = 4)	-	313.4 ± 417.5 (n = 3)	-	p = 0.4795
At 4 weeks	387.3 ± 165.4 (n = 3)	p = 0.1088	662.0 ± 619.4 (n = 2)	p = 0.1797	p = 0.5637
Serum LDH, mean ± SD, U/L					
At start of maintenance treatment	332.5 ± 84.1 (n = 2)	-	303.0 ± 86.0 (n = 3)	-	p = 0.5637
At 4 weeks	243.0 ± 32.5 (n = 2)	-	273.5±71.4 (n = 2)	p = 0.6547	p = 0.4386
Serum TARC, mean ± SD, pg/mL					
At start of maintenance treatment	504.0 ± 238.5 (n = 4)	-	918.0 ± 664.2 (n = 3)	-	p = 0.4795
At 4 weeks	703.0 ± 256.6 (n = 3)	p = 0.1088	1689.0 ± 1156.8 (n = 2)	p = 0.1797	p = 0.2482
Corticosteroid-related parameters					
Serum cortisol, mean ± SD, µg/dL					
At start of maintenance treatment	3.13 ± 1.64 (n = 3)	-	6.24 ± 1.08 (n = 2)	-	p = 0.0832
At 4 weeks	3.13 ± 1.38 (n = 2)	p = 0.1797	4.87±2.07 (n = 2)	p = 0.6547	p = 0.4386
ACTH, mean ± SD, pg/mL					
At start of maintenance treatment	18.7 ± 12.7 (n = 2)	-	28.4 ± 8.7 (n = 2)	-	p = 0.4386
At 4 weeks	8.4 (n = 1)	-	16.7 ± 2.0 (n = 2)	p = 0.1797	p = 0.2207

p†: Wilcoxon signed rank-sum test, p‡: Wilcoxon rank-sum test.

Abbreviations: ACTH, adrenocorticotropic hormone; IgE, immunoglobulin E; LDH, lactate dehydrogenase; SD, standard deviation; TARC, thymus and activation-regulated chemokine.
