# Supplementary material for: Proactive versus Rank-Down Topical Corticosteroid Therapy for Maintenance of Remission in Pediatric Atopic Dermatitis: A Randomized, Open-Label, Active-Controlled, Parallel-Group Study (Anticipate Study)
Source: J Clin Med. 2022 Oct 31;11(21):6477. doi: 10.3390/jcm11216477 (PMC9658234; doi:10.3390/jcm11216477)
Supplement: Supplementary file 1 [file jcm-11-06477-s001.zip › SuppInfo_Supplemental Table S2.pdf]

Supplemental Table S2. Blood test results: Individual data.

	Total IgE (IU/mL)	Peripheral blood eosinophil count (/μL)	LDH (U/L)	TARC (pg/mL)	Cortisol (μg/dL)	ACTH (pg/mL)
Proactive therapy group						
Patient 1						
At start of maintenance treatment	2466.2	441.0	—	856	2.7	—
At 4 weeks	—	504.0	220	996	4.1	—
Patient 2						
At start of maintenance treatment	626.7	136.8	—	444	—	—
At 4 weeks	—	198.0	—	518	—	—
Patient 3						
At start of maintenance treatment	20.0	420.0	273	375	1.75	9.7
At 4 weeks	—	460.0	266	595	2.15	8.4
Patient 4						
At start of maintenance treatment	3280.0	1050.0	392	341	4.94	27.7
At 4 weeks	—	—	—	—	—	—
Rank-down therapy group						
Patient 5						
At start of maintenance treatment	4310.0	790.0	346	1646	5.48	22.2
At 4 weeks	—	1100.0	324	2507	6.33	15.3
Patient 6						
At start of maintenance treatment	370.0	12.3	359	345	—	—
At 4 weeks	—	—	—	—	—	—
Patient 7						
At start of maintenance treatment	91.4	138.0	204	763	7.0	34.5
At 4 weeks	—	224.0	223	871	3.4	18.1

Abbreviations: ACTH, adrenocorticotropic hormone; IgE, immunoglobulin E; LDH, lactate dehydrogenase; SD, standard deviation; TARC, thymus and activation-regulated chemokine.
